# Announcements and Corrections

**Published:** 1914-09

**Authors:** 


### ANNOUNCEMENTS AND CORRECTIONS

---

The Hon. Henry W. Hill, editor in medical jurisprudence, states that important rulings have been made by the Attorney General of N. Y., regarding the new medical laws. These rulings are not yet accessible, hence we have published the laws—with certain omissions not of direct interest to the medical profession—and will publish rulings and comments upon the laws as soon as possible. It may be permissible to state that our publication of medical laws, read and heeded, would have saved the profession of Buffalo in fines, during the year just completed, more than the entire subscriptions for the entire profession of the city.

